# Quantifying Sweet Taste Liker Phenotypes: Time for Some Consistency in the Classification Criteria

**DOI:** 10.3390/nu11010129

**Published:** 2019-01-10

**Authors:** Vasiliki Iatridi, John E. Hayes, Martin R. Yeomans

**Affiliations:** 1 School of Psychology, University of Sussex, Falmer BN1 9QH, UK; martin@sussex.ac.uk; 2 Department of Food Science, College of Agricultural Sciences, The Pennsylvania State University, University Park, PA 16802, USA; jeh40@psu.edu; 3 Sensory Evaluation Center, College of Agricultural Sciences, The Pennsylvania State University, University Park, PA 16802, USA

**Keywords:** sweet taste, hedonics, sweetness, taste test, individual differences, classification method

## Abstract

Taste hedonics is a well-documented driver of food consumption. The role of sweetness in directing ingestive behavior is largely rooted in biology. One can then intuit that individual differences in sweet-liking may constitute an indicator of variations in the susceptibility to diet-related health outcomes. Despite half a century of research on sweet-liking, the best method to identify the distinct responses to sweet taste is still debated. To help resolve this issue, liking and intensity ratings for eight sucrose solutions ranging from 0 to 1 M were collected from 148 young adults (29% men). Hierarchical cluster analysis (HCA) revealed three response patterns: a sweet-liker (SL) phenotype characterized by a rise in liking as concentration increased, an inverted U-shaped phenotype with maximum liking at 0.25 M, and a sweet-disliker (SD) phenotype characterized by a decline in liking as a function of concentration. Based on sensitivity and specificity analyses, present data suggest the clearest discrimination between phenotypes is seen with 1.0 M sucrose, where a liking rating between −15 and +15 on a −50/+50 scale reliably distinguished individuals with an inverted U-shaped response from the SLs and the SDs. If the efficacy of this approach is confirmed in other populations, the discrimination criteria identified here can serve as the basis for a standard method for classifying sweet taste liker phenotypes in adults.

## 1. Introduction

Hedonic responses to taste stimuli are dissociable construct from motivation or a desire to eat (i.e., “liking” vs. “wanting”) as proposed by Berridge [1], and these responses influence dietary intake [2,3,4]. Elsewhere, a conceptual model linking sensation to intake via affective/hedonic responses has also been proposed [5]. Under these models, it is highly plausible that interpersonal variations in hedonic responses to sweet taste—in conjunction with genetic and epigenetic inputs, environmental forces, and other acquired individual characteristic—may contribute to variations in the susceptibility for obesity and obesity-related diseases. For almost half a century, observations of distinct individual liking patterns to sweet taste stimuli have repeatedly been made, thereby challenging the widespread belief that sweetness is universally highly liked. Witherly and colleagues, for example, speculated that humans exhibit up to four distinguishable responses to various sweetened beverages [6], which, as was also illustrated later by Drewnowski [7], could be described as a rise in liking with increasing sweetener concentration followed by a decline (Type I), a rise and then a plateau (Type II), a monotonic decline (Type III), and a non-systematic change in liking (Type IV).

**Figure 1:**
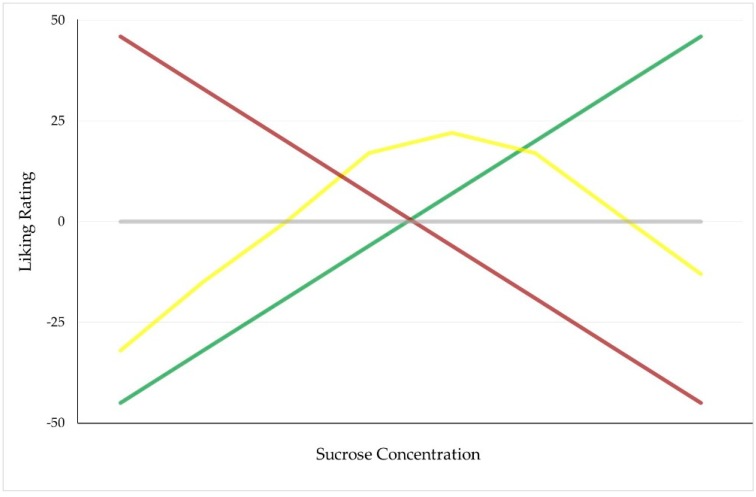
Graphical representation of the most commonly reported sweet taste liker phenotypes. The green line corresponds to a phenotype characterized by a rise in liking with increasing sucrose concentration (e.g., sweet liker phenotype), yellow line illustrates an inverted U-shaped hedonic response as a function of sucrose concentration (e.g., inverted-U phenotype), grey line represents an insensitive response to changes in sucrose concentration, and red line corresponds to a phenotype characterized by a decline in liking as sucrose concentration increases (e.g., sweet disliker phenotype). Adapted with permission from Reference [11].

Since the pioneering work of Pangborn [8], sensory scientists using simple sucrose solutions and multiple different scaling methods in laboratory settings have similarly identified at least four different sweet taste liker phenotypes. As summarized in Figure 1, the associated response patterns are characterized by either a positive slope, a horizontal (“flat”) slope, an inverted U-shape, or a negative slope. Simpler schemes also exist, where participants are dichotomized into sweet likers (SLs) and sweet dislikers (SDs). The SL phenotype (sometimes reported as the Type II phenotype) generally refers to liking for ever-higher sweetness (e.g., in References [9,10]) and accounts for 48.5% of the published literature [11]. In contrast, the SD phenotype, which shares a very similar distribution (48.2%) with the SL phenotype [11], has been defined differently across various studies: it can describe either as a monotonically decreasing liking as sucrose concentration increases (e.g., in References [12,13]), or a liking for moderate levels of sweetness, which is graphically presented as an inverted U (e.g., in Reference [14]) and sometimes also called Type I phenotype (e.g., in References [15,16]). To note, a few studies identifying both subtypes of the SD response pattern classified them into a single group reported as SD phenotype, as well (e.g., in References [17,18]).

Accordingly, an important question to be addressed is how these distinct hedonic responses to sweet taste can be defined and identified. Among 71 studies we recently reviewed [11], four main phenotyping methods (each relying on different classification criteria) were identified: the visual or algorithmic interpretation of hedonic responses from multiple sucrose concentrations (Method 1a and Method 1b, respectively), the “highest preference using ratings” method that dichotomizes participants based on whether they like the highest sucrose concentration tested the most (Method 2), the “average liking above mid-point” or “positive/negative liking” method where liking ratings are compared to one or two predefined cut-off scores (Method 3), and the “highest preference via paired comparisons” method that categorizes participants based on which sucrose concentration they prefer the most (Method 4). As detailed in our recent review [11], Method 2 and Method 3 suffer from arbitrariness associated with the strength of the taste stimuli and/or the classification rating thresholds, and both methods are prone to misclassification. The dependence on visual inspection in Method 1a raises the potential for subjective interpretation, and Method 4 involves a choice paradigm based on preference rather than liking per se.

Considering these methodological challenges, along with the ongoing debate over the role of sugar intake as a factor in obesity [19,20,21,22], there is strong need for a more precise and consistent method to identify sweet taste phenotypes. The numerous prior studies that have investigated the presence of different sweet taste liker phenotypes and their potential relationship to dietary intake (e.g., in References [14,18,23]) or to body mass index (BMI: e.g., in References [13,16,24,25,26]) have used widely different methods to define phenotypes; presumably, this has contributed to the inconsistencies reported across studies. Accordingly, in our recent review [11], we suggested that a rapid and reliable phenotyping method is needed to facilitate comparisons across future studies. In our review, we proposed that an optimal sucrose concentration be identified that best separates distinct sweet taste liker phenotypes, in terms of sensitivity and specificity. In 2015, Asao et al. [27] piloted this idea in order to discriminate SLs from SDs. However, as commonly happens with small pilot studies, their sample size likely affected the phenotyping process, potentially leading to an underestimation of the true number of distinct response patterns, a limitation the authors noted in their report. Further, the total number of stimuli they used was rather large [27], raising additional issues of fatigue, adaptation, and inattentiveness. Finally, their participants were tested after they had fasted for an average of 12.1 h [27], which may influence the appetitiveness of the stimuli.

The present study aimed to extend the approach used by Asao et al. [27] while also eliminating some of the methodological issues mentioned above toward a goal of defining a new standardized phenotyping method. We had three aims. First, we identified different sweet taste liker phenotypes statistically. Second, we analyzed these phenotyping data to identify a single sucrose concentration where an application of one or two specific cut-off liking scores ensures the most reliable and replicable definition of each of the identified phenotypes. Last, potential relationships between the motivational state and baseline characteristics of our participants with these sweet taste liker phenotypes were explored.

## 2. Materials and Methods

### 2.1. Participants

A total of 148 non-diabetic participants aged 18–34 were recruited from students and staff at the University of Sussex between September and December 2017 (Table 1). Cohort size was determined by the suggested minimum of 100 participants in our recent methodological review for the successful identification of the main sweet taste liker phenotypes [11], which was further increased to adjust for the expected underrepresentation of the SD phenotype in our young adult population. Inclusion criteria comprised being medication free (other than oral contraception), smoking less than five cigarettes a week, and having no history of diagnosed eating disorders. Individuals with a current respiratory illness or having recently (less than two weeks) undergone a dental procedure, those being on a weight loss or a medically induced special diet, and women with an irregular menstrual cycle were also excluded. At enrollment, participants gave their written informed consent for inclusion in the study, but they were naive to the study’s hypothesis until they had completed all tasks (debriefing provided). The University of Sussex Science and Technology Cross-Schools Research Ethics Committee approved the protocol on the 22 September 2017 (ER/VI40/1), and the study was conducted in accordance with the 1964 Declaration of Helsinki.

### 2.2. Taste Test

#### 2.2.1. Taste Stimuli

To ensure sufficient individual ratings for the development of hedonic curves while trying to minimize confounding effects of adaptation [28] and sensory specific satiety [29], the taste test consisted of seven different aqueous sucrose solutions (0.03125, 0.0625, 0.125, 0.25, 0.5, 0.67, and 1 M) and one water blank, replicated in two separate blocks, for a total of 16 tastings.

The particular concentration range tested was equivalent to sucrose solutions between 1.07% and 34.23% (*w/v*) based on density at 20 °C [30], and were chosen to reflect four different considerations: (1) previously reported effects of age on sucrose recognition thresholds [31,32,33]; (2) the most commonly used sucrose concentrations in prior relevant studies (reviewed in Reference [11]); (3) the sweetness typically encountered in sugar-sweetened beverages [34]; and (4) a compromise between equal log spacing and serial dilution for sample preparation. 

All sweet stimuli were prepared at least 24 hours in advance by dissolving food-grade sucrose in mineral water at room temperature. Solutions were stored at 4 °C until used. On the experimental day, solutions were allowed to warm up to room temperature prior to presentation, and were presented as 10 mL samples in transparent 60 mL glass cups labelled with random three digit codes. For the solute and rinsing, we used a commercial mineral water with the lowest dry residue concentration available at the time (Volvic, Danone Waters London and Ireland Ltd., London, U.K.). 

#### 2.2.2. Rating Scales

Participants evaluated liking and intensity for each stimulus using a horizontal visual analogue scale (VAS) end-anchored with “dislike extremely” (scored −50) and “like extremely” (scored +50) and a vertical generalized labeled magnitude scale (gLMS) with properly positioned descriptors ranging from “no sensation” (scored 0) to “strongest imaginable sensation of any kind” (scored +100), respectively. To ensure within and between-subjects compliance, training for both scales was provided. The practice session for VAS involved rating liking for a series of non-food items, while training in the use of gLMS was applied by evaluating responses to noise and light [35].

On the basis of Cabanac’s theory regarding possible enhancement of stimulus value by internal state (“alliesthesia” [36]), two series of VAS appetite ratings [37] were completed before the first and after the second taste test block. All ratings were collected using the Sussex Ingestion Pattern Monitor (SIPM version 2.0.13, University of Sussex, Falmer, U.K.), a computer-based system developed to record and score rating data.

#### 2.2.3. Procedure

The taste test was conducted approximately 2 h after breakfast (between 09.30 am and 12.30 pm depending on each participant’s personal routine). Participants were also asked to abstain from smoking, chewing gum, and tooth brushing for the 2 h prior to testing; no restrictions applied to water consumption. During both taste test blocks, a “sip and spit” protocol was followed: participants were instructed to place the entire 10-mL solution in their mouth, swirl it around for 10 s, and expectorate it. They then rated their liking and sweetness intensity before rinsing their mouth with water and proceeding to the next sample. Stimuli were presented in randomized order with participants blinded to the concentration of sucrose tasted each time. After the taste test was complete, demographic (date of birth, sex, and ethnicity) and lifestyle characteristics (“Have you ever been on a diet in order to lose weight?” with possible answers “Yes, one or more times in the past” or “Never,” and “Did you usually add more sugar in your coffee, tea or cereals when you were younger?” with possible answers “Yes, I used to add more sugar in my coffee, tea or cereals when I was younger,” or “No, I add the same sugar as I did in the past,” or “Never added sugar in my coffee, tea or cereals”) were collected.

### 2.3. Anthropometry

To minimize any possible interactions between the sensory ratings and anthropometric measures, participants revisited the laboratory for a separate early morning session (08:30–10:30) for anthropometry; this visit was scheduled between two days and two weeks after the taste test. Height was measured to the nearest 0.1 cm using a stadiometer and weight to the nearest 0.1 kg using a calibrated body composition analyzer (MC-780MA P, TANITA, Tokyo, Japan). Standardized procedures were followed, including wearing light clothing and no shoes [38].

### 2.4. Statistical Analysis

Our primary goals were to (a) algorithmically identify the different sweet taste liker phenotypes in our study cohort and (b) to determine the specific sucrose concentration and associated cut-off score(s) for liking ratings that most reliably allowed for the identification of those distinct phenotypes. Assumptions of normality were tested prior to the main statistical analyses using visual inspection (histograms, Q-Q plots, and bloxplots), and summary statistics (skewness and kurtosis *z*-scores computed by dividing skewness or kurtosis values with the associated standard errors). *Z*-scores (absolute values) larger than 1.96 were indicative of a normal distribution. All ratings are reported as means and standard errors (normally distributed), while medians and ranges are used for age and BMI (not normally distributed); categorical characteristics are expressed as percentages. 

Interclass correlation coefficients (ICCs) were calculated to assess test–retest reliability of liking ratings over the two taste test blocks. Given our experimental design, an average measures absolute agreement two-way mixed-effects model was selected [39]. Per the guidelines, an ICC value less than 0.5 indicates poor reliability, values between 0.5 and 0.75 reflect moderate reliability, and values between 0.75 and 0.9 indicate good reliability [40].

As the first step to achieve the principle aim of the current study, an agglomerative hierarchical cluster analysis (HCA) was performed and meaningful groups (clusters) of participants who shared similar liking patterns within each group but were heterogeneous in the between-group contrasts were identified. The mean liking ratings from the eight replicated concentrations in the two taste test blocks were treated as the dimensions for the HCA. The squared Euclidean distance between pairs of cases or clusters and the between-groups (average) linkage method were selected to assist with the merging process [41]. The final decision on the true number of clusters in our dataset was dictated graphically by interpreting the scree plot of coefficients of the agglomeration schedule we designed (Office Excel 2013 for Windows, Microsoft, Washington, DC, USA) and then applying this information (“stopping rule”) to the dendrogram provided by the statistical software on the HCA output [41].

We then implemented a two-by-two cross tabulation function to estimate the dyads of sucrose concentration and liking score with the highest sensitivity and specificity in predicting the three distinct sweet taste liker phenotypes. In each two-by-two cross tabulation table, the phenotyping results emerged when a specific dyad of sucrose concentration and liking score was used as the classification criteria for the identification of the sweet taste liker phenotype under investigation were contrasted with the associated phenotyping results suggested by the HCA. The number of true positives (e.g., classified as SL by both the dyad tested and the HCA) and the number of true negatives (e.g., not classified as SL by both the dyad tested and the HCA) indicated the sensitivity and specificity attached to that particular dyad of sucrose concentration and liking score, respectively. Reported liking ratings for stimuli from 0.03125 M to 1.0 M sucrose and potential cut-off values ranging between −20 and +20 in 5-point increments were tested for their prediction value. A K-1 series of sensitivity-specificity tests were conducted, where k represents the number of main clusters previously identified in the HCA.

To test the hypothesis that the sucrose concentration (within subject factor) and the initial clusters or subsequent sweet taste liker phenotypes (between subject factor), as well as their interaction, affect liking and intensity ratings of the presented sweet taste stimuli, two-way mixed ANOVAs with Greenhouse-Geisser correction were carried out. We also employed separate one-way ANOVAs to contrast liking and intensity (both mean ratings and ratings across each of the eight concentrations) by sweet taste liker phenotype. In cases of violation of the equal variances assumption, Brown–Forsythe tests were applied, instead [42]. Post hoc Fisher’s least significant difference (LSD) and Games-Howell tests were used as appropriate to further understand the nature of specific paired comparisons.

Nonparametric (Mann–Whitney) tests for the previously reported not normally distributed continues variables (age and BMI) and Pearson’s chi-square tests for the categorical variables (gender, ethnicity, dieting history, and habitual use of table sugar) were used to investigate for differences in participant characteristics across the distinct sweet taste liker phenotypes. To explore whether there were also gender differences in measures of interest, additional chi-square tests were performed. Phi symmetric measures instead of Pearson’s results are reported in cases of cells with an expected count less than 5.

To ensure participants’ compliance with the taste test protocol, changes in hunger and thirst before and after delivering the taste test were explored using paired *t*-tests. We also calculated multiple linear regressions to investigate the degree to which pre- and post-test hunger and thirst predicted liking and intensity ratings across the study sample. The influence of pre- and post-test levels of hunger and thirst was further explored using either one-way ANOVAs or Brown–Forsythe tests [42] to detect differences across the distinct sweet taste liker phenotypes.

The extent to which our method for the identification of the distinct sweet taste liker phenotypes agrees with those in previous literature (see Introduction for details) was assessed by Cohen’s Kappas and 95% confidence intervals (CIs) based on the “Estimate ± 1.96 × Standard Error” formula [43]; participants exhibiting an inverted U-shaped response were excluded from this analysis due to the bimodal nature of the phenotyping results elicited by Method 2 and 3. The relevant frequency distributions were also estimated. For the comparison with Method 2 participants who rated the highest sucrose concentration, namely the 1 M solution, as the most pleasant were considered as SLs, whilst all remainder liking patterns were classified into the SD phenotype [44,45]. The agreement with Method 3 was tested using the 0.5 M sucrose solution and the corresponding neutral cut-off hedonic score of 0 (zero) as the classification criteria to discriminate SLs from SDs [23].

Unless otherwise stated, data were analyzed using SPSS Statistics for Windows, version 24.0 (IBM Corp., Armonk, NY, USA). An alpha level of 0.05 was set as the threshold for statistical significance and all performed statistical tests were two-tailed. 

## 3. Results

### 3.1. Participant Characteristics

Participant characteristics are summarized in Table 1; three (two women and one man) failed to report to the laboratory for both sessions. As a whole the cohort tested here was relatively young and lean (*Mdn* = 20.2 years and *Mdn* = 22.1 kg/m^2^, respectively) and was mainly comprised of women (70.9%); most self-identified as Caucasian (75.7%). Nearly half of the participants reported that they currently add less sugar in their drinks and cereals than when they were younger, and one in three had been on a diet for weight loss at least once in the past. Overall, the women were slightly younger than the men (*Mdn* = 21.1 years for men and *Mdn* = 20.1 years for women; *U* = 1454.5, *Z* = −3.263, *p* = 0.001), and had a lower average BMI (*Mdn* = 23.4 kg/m^2^ for men and *Mdn* = 21.6 kg/m^2^ for women; *U* = 1475.5, *Z* = −2.861, *p* = 0.004). This was expected, as it reflects the typical differences in BMI between men and women and the differences in BMI across different age groups in the U.K. [46].

### 3.2. Taste Test

**Figure 2:**
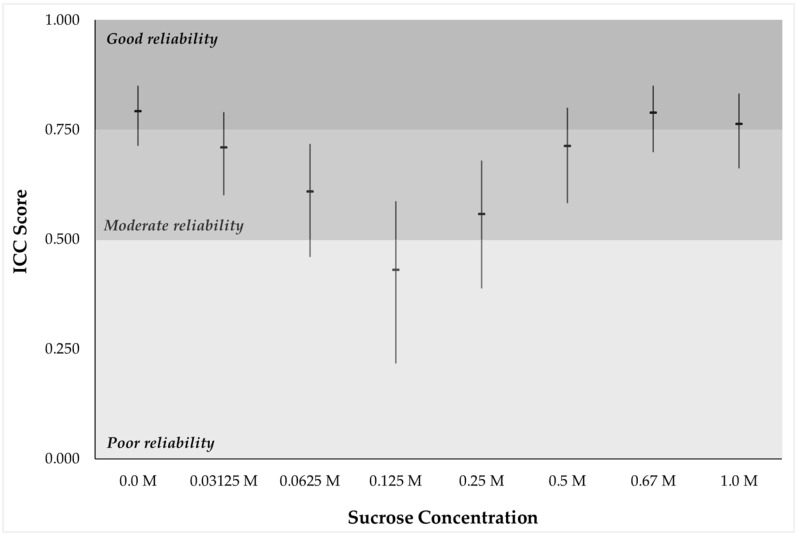
Interclass correlation coefficient (ICC) scores (95% confidence interval) for liking ratings from the two taste test blocks across the different taste stimuli.

Test-retest reliability analysis comparing liking ratings across the two taste test blocks indicated moderate to good reproducibility based on the ICC cut-offs suggested by Portney and Watkins [40] for all but the 0.125 M solution (Figure 2). The two highest sucrose concentrations (0.67 and 1.0 M), and water were associated with the strongest agreement between the two repetitions. As expected, there was a main effect of concentration on liking across all participants with significantly different mean hedonic scores reported for different solutions (*F*(2.12, 312.15) = 10.65, *p* < 0.001, *ηp*^2^ = 0.068).

#### 3.2.1. Identifying Distinct Responses to Sweet Taste: HCA

HCA resulted in ten subgroups of distinct responses to sweet taste with a significant effect of cluster on liking (*p* < 0.001 for all eight sucrose concentrations and effect sizes ranged from 0.22 for the 0.125 M solution to 0.80 for the 1.0 M solution). Three main clusters that accounted for 92% of the study sample were observed. Cluster 1 (*n* = 44) and cluster 3 (*n* = 22) described hedonic response patterns consistent with the sweet liker (SL) and sweet disliker (SD) phenotypes. Both trends were particularly evident for solutions with added sucrose above 0.125 M. Notably however, almost half of the study sample fell into cluster 2 (*n* = 70), where liking increased modestly with concentration up to an intermediate level of sucrose (0.25 M) and then decreased as the concentration continued to increase. Remarkably, participants who were classified into cluster 2 rated both the lowest (*M* = 1.0, *SEM* = 0.76 for 0.03125 M) and the highest (*M* = −1.5, *SEM* = 1.44 for 1.0 M) sucrose concentration as neutral; that is, they neither liked them nor disliked them (*t*(69) = 1.46, *p* = 0.148 for the paired comparison between the lowest versus the highest concentration).

Regarding the 12 participants classified into one of the remaining clusters (clusters 4 to 10), plotting liking as a function of concentration revealed that participants in cluster 9 (*n* = 2) and those in cluster 10 (*n* = 3) followed a classical SL and a SD liking pattern, respectively. Their ratings from the eight different sucrose concentrations resulted, however, in steeper liking curves (“extreme” responses) than those in our main SL and SD clusters, which explains why they emerged as separate groups during the clustering procedure. Indeed, before we applied the “stopping rule” as appropriate (see Section 2.4 for details), participants grouped into clusters 9 and 10 and those grouped into clusters 1 and 3, respectively, had been considered as homogenous only subsequent to the inverted U-shaped phenotype merged with the SL phenotype. Likewise, an inverted U-shaped response corresponding to corresponding to that of cluster 2 was observed for participants classified into cluster 4 (*n* = 2), cluster 7 (*n* = 2), and cluster 8 (*n* = 1): among the heterogeneous mean liking ratings to those of cluster 2, a different optimal sweetness (0.5 M for cluster 4 and 0.67 M for cluster 8) and a higher rating for the breakpoint concentration of 0.25 M sucrose (*M* = 8.9, *SEM* = 1.15 for cluster 2 and *M* = 28.5, *SEM* = 4.50 for cluster 7, *t*(70) = −2.84, *p* = 0.006) stand out. Two single cases of erratic responses were also identified and eliminated from further analysis (cluster 5 and cluster 6).

#### 3.2.2. Identifying Distinct Sweet Taste Like Phenotypes: New Classification Criteria

With regard to the specific sucrose concentration and liking thresholds that best discriminated between the three main clusters, the 1 M solution and liking scores of −15 or lower for the identification of SDs and +15 or higher for the identification of SLs were associated with the lowest number of false negative classifications (90.9 and 97.7 percentage sensitivity for SDs and SLs, respectively) and the lowest number of false positive classifications (93.9 and 93.5 percentage specificity for SDs and SLs, respectively). The results are shown in Table 2 and Table 3.

We then applied these classification criteria individually to participants who were assigned to the remaining clusters. The revised grouping (SL phenotype: *n* = 46; 31.5%, inverted U-shaped phenotype: *n* = 73; 50%, SD phenotype: *n* = 27; 18.5%) was in agreement with the classification suggested by the visual interpretation of the shape of the relevant liking curves in all participants except those initially classified into cluster 4. Those participants met the new SD phenotype criteria rather the criteria associated with the inverted U-shaped response pattern. A closer inspection of their hedonic responses revealed that they actually had rated all sucrose solutions as neutral or unpleasant. In addition, integrating the very small clusters into the main groups of responses reduced overfitting and allowed for the subsequent statistical analyses required.

**Figure 3:**
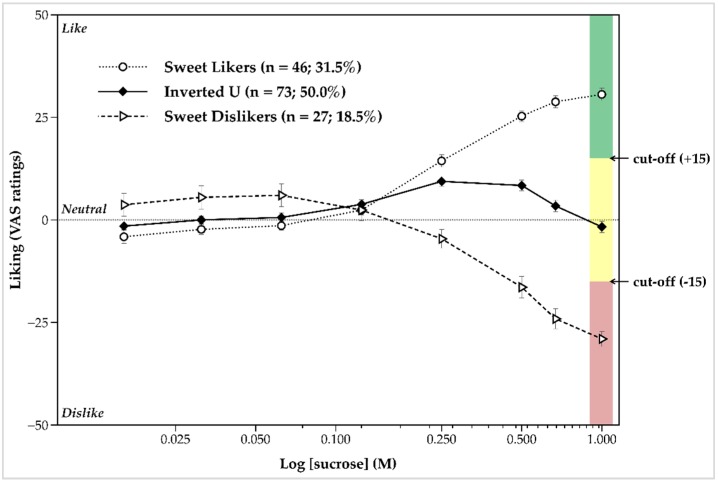
Liking ratings (mean ± standard error of the mean) as a function of sucrose solutions by the three sweet taste liker phenotypes. Ratings were averaged across the two taste test blocks. The response pattern for the sweet liker phenotype is displayed with a dotted line, the response pattern of inverted U-shaped phenotype with a solid line, and the response pattern of sweet disliker phenotype with a dashed line. Different colors denote the different ranges of liking ratings for 1 M sucrose which, according to the relevant sensitivity and specificity checks (see Table 2 and Table 3 for details), could be used for the reliable discrimination between the three distinct sweet taste liker phenotypes: green color corresponds to the range of liking ratings for 1 M sucrose representing sweet likers, yellow color indicates the hedonic response spectrum to 1 M sucrose characteristic of the inverted U-shaped phenotype, and red color corresponds to the range of liking ratings for 1 M sucrose for sweet dislikers.

Confirming the diverse nature of the sensory responses to sweet taste among participants classified into the three main sweet taste liker phenotypes, overall liking and intensity significantly varied across these newly defined distinct groups, *F*(2, 56.21) = 89.44, *p* < 0.001 for liking and *F*(2, 77.95) = 5.74, *p* = 0.005 for intensity. A main effect of sucrose concentration (*F*(4.44, 635.19) = 8.53, *p* < 0.001, *ηp*^2^ = 0.056), as well as a strong interaction effect between sucrose concentration and phenotype (*F*(8.88, 635.19) = 78.65, *p* < 0.001, *ηp*^2^ = 0.524) on liking were also found. As shown in Figure 3, follow-up analysis indicated that participants with an inverted U-shaped response liked the three lower sucrose concentrations at a similar level when compared with both SLs and SDs. When liking ratings of those stimuli were separately contrasted between the two extreme phenotypes, we found that SLs rated them as less pleasant than SDs did. Liking for the 0.125 M sucrose solution did not differ between groups, whereas liking ratings for the rest of the sweet taste stimuli significantly differed by cluster (*p* < 0.001 for most paired comparisons).

**Figure 4:**
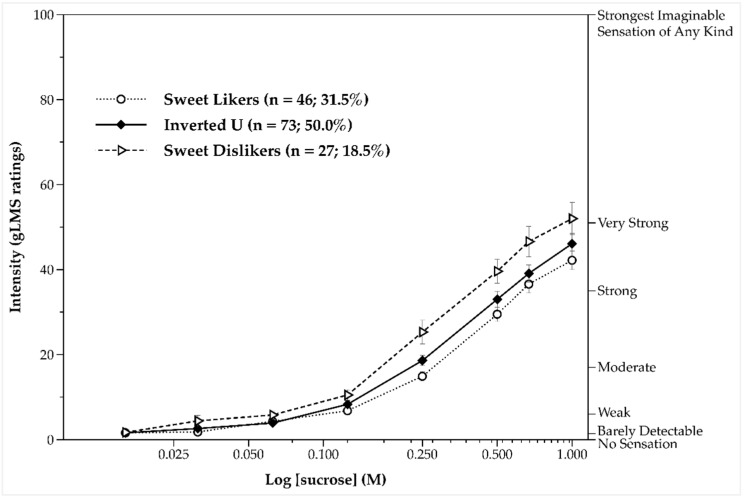
Intensity ratings (mean ± standard error of the mean) as a function of sucrose solutions by the three sweet taste liker phenotypes. Ratings are averaged across the two taste test blocks. The intensity curve of the sweet liker phenotype is displayed with a dotted line, the intensity curve of the inverted U-shaped phenotype with a solid line, and the intensity curve of the sweet disliker phenotype with a dashed line.

We next sought to examine the perceived variations in sweetness for the different stimuli between the three sweet liker phenotypes. Paired comparisons between the intensity ratings for each successive concentration and the intensity ratings for the previous indicated that participants were clearly able to distinguish between the different sucrose concentrations (*p* = 0.002 for water and 0.03125 M, and *p’s* < 0.001 for all remainder pairs). Rated intensity also increased as sucrose concentration increased across all three sweet taste like phenotypes, *F*(2.32, 336.30) = 535.25, *p* < 0.001, *ηp*^2^ = 0.787 (Figure 4). SDs overall perceived the taste stimuli as sweeter (*M* = 23.3, *SEM* = 1.62) than both SLs (*M* = 17.2, *SEM* = 0.73; *p* = 0.001) and participants classified in the inverted U-shaped phenotype (*M* = 19.2, *SEM* = 0.96; *p* = 0.015). No interaction effect between concentration and sweet taste like phenotype on intensity was, however, observed, *F*(4.67, 333.68) = 521.10, *p* = 0.082, *ηp*^2^ = 0.027.

**Figure 5:**
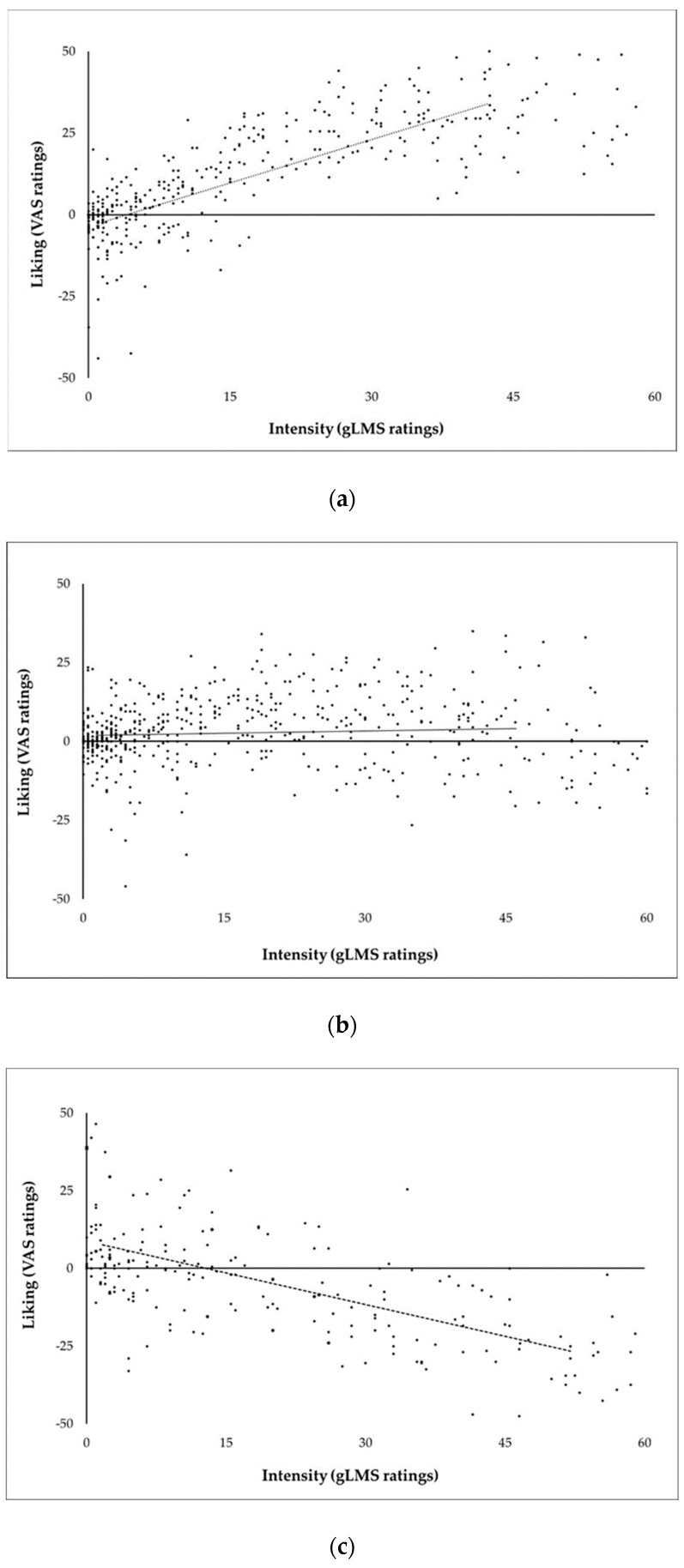
Individual ratings of liking as a function of perceived intensity for the sweet taste stimuli in (**a**) sweet likers, (**b**) individuals exhibiting an inverted U-shaped hedonic response, and (**c**) sweet dislikers. Lines represent the average ratings across individuals classified within each phenotype.

To explore whether the identified sweet taste liker phenotypes were merely indirect consequences of differences in perceived intensity rather than true differences in hedonics per se, liking ratings were also plotted as a function of intensity separately for the three main clusters. As shown in Figure 5a–c, no such indication was found.

#### 3.2.3. Pre- and Post-Test Levels of Hunger and Thirst

Pre-test levels of hunger (*M* = −7.5, *SEM* = 2.11) and thirst (*M* = 0.3, *SEM* = 1.68) confirmed participants’ compliance with the taste test preparation instructions, whereas the increase in hunger (*t*(147) = −3.25, *p* = 0.001) and decrease in thirst (*t*(147) = 2.32, *p* = 0.022) over time was also in line with the effects of the “sip and spit” and “mouth rinsing with water” parts of the taste protocol. Neither hunger nor thirst ratings before taste test block 1 or after taste test block 2 predicted liking (*F*(2, 145) = 2.065, *p* = 0.130 for pre-test levels of hunger and thirst; *F*(2, 145) = 0.607, *p* = 0.546 for post-test levels of hunger and thirst) or intensity (*F*(2, 145) = 1.041, *p* = 0.356 for pre-test levels of hunger and thirst; *F*(2, 145) = 0.403, *p* = 0.669 for post-test levels of hunger and thirst) across the study sample. When ratings of hunger and thirst were examined against the three distinct sweet taste liker phenotypes, non-significant differences were found (*F*(2, 143) = 2.410, *p* = 0.093, and *F*(2, 143) = 0.094, *p* = 0.910 for pre-test levels of hunger and thirst, respectively; *F*(2, 76.22) = 0.986, *p* = 0.378, and *F*(2, 143) = 0.107, *p* = 0.899 for post-test levels of hunger and thirst, respectively). These data clearly show that the group differences in sweet liking cannot be attributed to the observed changes in hunger or thirst.

### 3.3. Participant Characteristics by Sweet Taste Liker Phenotype

Possible variations in participant characteristics relative to sweet taste liker phenotype were also examined. Gender (*χ*^2^(2, *N* = 146) = 2.39, *p* = 0.302), ethnicity (*φ* = 0.152, *p* = 0.496), dieting history (*χ*^2^(2, *N* = 144) = 1.84, *p* = 0.400), habitual use of table sugar (*φ* = 0.194, *p* = 0.240), age (*H*(2) = 2.60, *p* = 0.273) and BMI (*H*(2) = 0.67, *p* = 0.717) did not differ between groups. All associated values by phenotype are summarized in Table 1.

### 3.4. Comparison to Existing Classification Methods

**Figure 6:**
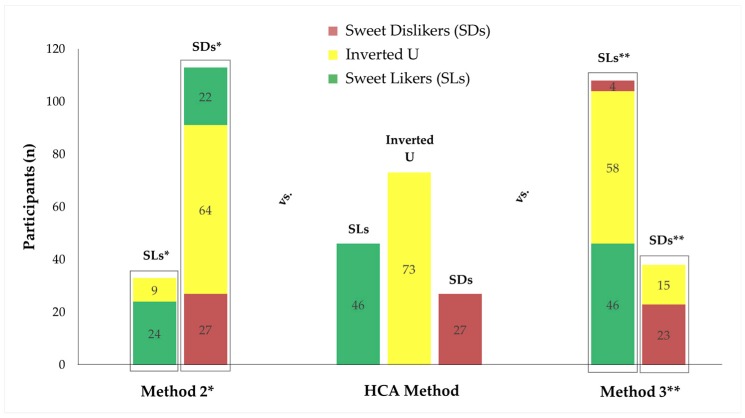
Comparison of the distribution of sweet taste liker phenotypes in our study sample when different classification methods were used. Method 2 (rating the 1 M sucrose solution or not as the most pleasant) and Method 3 (rating the 0.5 M sucrose solution higher than 0 or not) were, by definition, limited to a two-response group phenotyping outcome (binomial distribution), while HCA method (rating the 1 M sucrose solutions higher than +15, lower than −15, or between −15 and +15) allowed for the identification of three distinct sweet taste liker phenotypes. 133 participants (77.4%) versus 27 (18.5%) were classified as SDs and 108 participants (74.0%) versus 46 (31.5%) were classified as SLs when Method 2 and Method 3 were contrasted with the method we proposed here (HCA method), respectively. Different colors of the stacked columns and the associated data labels (numbers) correspond to the number of participants classified into the phenotype of the same color when the HCA method was used. Data labels (numbers) within each column add up to the total number of participants classified into the phenotype illustrated at the upper end of the relevant column. Asterisks (*/**) denote alternatives to our definition for SLs and SDs. SDs, sweet dislikers; SLs, sweet likers.

When Method 2 (rating the 1 M sucrose solution or not as the most pleasant) and Method 3 (rating the 0.5 M sucrose solution higher than 0 or not) were used to distinguish the different sweet taste liker phenotypes, the proportions of SD and the SL were respectively overestimated: 113 participants were classified as SDs according to Method 2 and 108 as SLs according to Method 3. Compared with our phenotyping method, in both cases, the majority of those participants (56.6% of SDs in Method 2 and 53.7% of SLs in Method 3) exhibited an inverted U-shaped response. Focusing on Method’s 2 phenotypic classification, all 27 participants classified as SDs using our method were also identified as SDs using Method 2. Regarding the SL phenotype, 22 out of 46 participants initially fell into the SL phenotype were classified as SDs using Method 2. Those 22 participants liked the 1 M sucrose solution significantly lower than the previous concentration (*M* = 25.3 for 1 M versus *M* = 30.6 for 0.67 M, *p* = 0.014), while no significant difference was observed when compared with the third higher sucrose concentration (*M* = 25.3 for 1 M versus *M* = 28.4 for 0.5 M, *p* = 0.222). The kappa coefficient was accordingly low at 0.447 (95% CI: 0.286 to 0.608). In contrast, the agreement with Method 3 was good with a Kappa coefficient at 0.879 (95% CI: 0.764 to 0.993). All SLs identified using our method were also classified as SLs by Method 3. The two phenotyping approaches were also in line regarding the SD phenotype: only four SDs using our method were discordantly classified as SLs using Method 3. Those participants had a mean liking for the 0.5 M barely over the neutral point (*M* = 1.1) and their liking rating for the 1 M, which was our concentration of choice for distinguishing sweet taste liker phenotypes, was as low as −28.7. A graphical representation of the level of consistency/disagreement among the methods compared here is provided in Figure 6.

## 4. Discussion

### 4.1. General Findings

The present report describes how hedonic responses to taste stimuli of varied sweetness can be algorithmically interpreted using HCA, and clustered into groups that represent similar sweet-liking patterns. For the current dataset, consistent differences in liking ratings across the eight sucrose solutions were found, which then allowed a clear characterization of participants as SLs, those with an inverted U-shaped response, or as SDs. Another key feature of the study was the subsequent identification of the 1 Μ aqueous sucrose solution and the VAS-based cut-off liking scores of −15 and +15 as the statistically reliable criteria to efficiently categorize individuals into these three different sweet taste liker phenotypes.

### 4.2. HCA Selection Advantages

Regarding our decision to use HCA for the identification of different sweet taste liker phenotypes, this was principally driven by the need for a statistically robust and unbiased merging of individuals into groups. Indeed, using an advanced statistical clustering technique allowed the three sweet taste liker phenotypes to emerge, whereas this would have been difficult to discern using more traditional visual inspection methods, particularly if the inspector was assuming a simple dichotomous mode. HCA is also based on hedonic responses across multiple stimuli rather than based on an arbitrarily selected single liking rating or the average value of hedonic scores of different stimuli. Accordingly, most elements of subjectivity and arbitrariness noted in the other phenotyping methods discussed earlier were controlled for. When we re-analyzed our current data using other widely used methods (defined as Methods 2 and 3 in the introduction, and in our recent review [11]), many participants were misclassified relative to the cluster analysis performed here, as the bimodal phenotyping approach in those methods assumes a priori that there are only two distinct response patterns. Critically, the HCA analysis shown here, as well as other recent studies [9,13], all suggest that response patterns for sweet stimuli are better described by three distinct phenotypes. Regarding the observed overestimation of SDs by Method 2 and of SLs by Method 3, this was a consistent feature of those methods in our recent evaluation of the impact of different sweet taste liker classification approaches [11]. In contrast, discriminating participants between the different sweet taste liker phenotypes based on a single sucrose concentration and predetermined cut-off liking scores as used in Method 3, led to the least misclassifications, further supporting the utility of such a phenotyping approach.

### 4.3. Phenotyping Results

Our findings confirm some [8,9,13,47,48] but not all, studies using phenotyping methods that allowed for a non-dichotomous identification of sweet-liking patterns. Indeed, in some published reports, participants with an inverted U-shaped response were considered as outliers [12,15,17], whilst elsewhere they were treated as homogeneous with the SDs [49,50,51]. Here, the generated icicle plot of our statistical output (not shown) revealed that during the final stages of the clustering process, SLs merged with those from the inverted U-shaped phenotype before SDs joined them both, uncovering a greater resemblance of the SL rather than of the SD phenotype to the inverted U-shaped response group. It is then plausible to assume that eliminating or misclassifying this intermediate phenotype is problematic and possibly obfuscates potential relationships between sweet taste liker phenotypes and health outcomes of interest. We also noticed that the sucrose concentration associated with the highest liking in the inverted U-shaped response group (i.e., the 0.25 M), was in line with the concentration observed in most previous work [15,16,17,18,27,52,53], although lower values have also been reported [8,14,48,54]. Practically speaking, this commonly identified 0.21–0.3 M range of sucrose concentration threshold in individuals who like intermediate levels of sweetness is lower than the sugars composition of the commercially available sweetened beverages [34]. This may potentiate the argument for reexamining the utility of sugar-tax policies [55]. The multisensory aspects of tasting real-life products should not, however, be disregarded [56], as well as the possible attenuating or enhancing effects of other flavor components on perceived sweetness in complex products [57,58,59,60]. As sagely noted by Pangborn, “a change in one ingredient can cause multiple physical-chemical interactions which alter several sensory attributes simultaneously: appearance, aroma, texture, taste etc.” [61] (p. 65).

Turning now to the frequency distribution of the identified sweet taste liker phenotypes, one third of our participants were classified as SLs, a proportion consistent with observations by others who also used HCA as their phenotyping method of choice [9,13,14]. Conversely, results in Asao et al. [27] and Kim et al. [62] indicate that this sweet-liking pattern accounted for roughly 50% of their study samples. Two possible explanations can be considered. First, the absence of a monotonically negative slope implies that individuals in both cohorts generally exhibited stronger liking for sweetness. Notably, in Kim et al. [62], two thirds of those classified in the inverted U-shaped phenotype rated 0.7 M as the most liked, a sucrose concentration breakpoint twice as high as the concentration we identified. Second, in those studies, sweet-liking was assessed under extreme motivational states with participants’ hunger [27,62] and/or satiety [62] being manipulated. Critically, when the same Korean researchers replicated their study using a more typical pretest protocol (i.e., refraining from eating for one to two hours prior to the taste test), their measures generally correspond with the data shown here. Focusing on the frequency distribution of the monotonically negative slope regardless of the SD label, our findings disagree with previous observations. For example, of the 650 age diverse adults tested by Garneau et al. [13], only 55 exhibited decreasing liking as concentration increased. Presumably, this is due to the relatively low sucrose concentrations they used; indeed, the highest concentration they used (0.40 M) fell near the concentration breakpoint we identified for our inverted U-shaped phenotype. In contrast, SDs in Kim et al. [9] were approximately as frequent as SLs and as participants in the inverted U-shaped phenotype (31.7, 32.5, and 35.8%, respectively). Nonetheless, they reported that, for the purposes of the study, two distinct clusters were treated as a single sweet-liking pattern representing the SD phenotype, with no further information provided; each of those clusters accounted for 10 and 21.7% of the total sample, respectively [9].

Here, despite the similar liking ratings of the lowest and the highest sucrose concentration by participants classified into the inverted U-shaped phenotype, perceived sweetness varied considerably when intensity ratings of those stimuli were contrasted. Therefore, this type of response cannot be attributed to reduced sensitivity to taste stimuli or from differences in recognition thresholds; rather, it appears to reflect a distinct liking pattern. Figure 5a,c indicated that this is also true for the SL and the SD phenotype, since inclusion of intensity ratings in the liking plots generated the expected liking patterns. In previous research, any differences in sweetness intensity between participants, when reported, were interpreted independent of the associated phenotyping results (e.g., in References [45,63,64]). The few studies that have contrasted sweetness intensity between the defined sweet taste liker phenotypes have had mixed outcomes: some studies report greater overall sweetness intensity in SDs than in SLs and/or than in other phenotypes in line with what we observed here [12,15,49,65], but the majority found no differences in sweet taste perception [10,13,16,66,67,68,69,70,71]. These inconsistencies could arise from several factors including the phenotyping methods and the stimuli concentrations used in these studies. Many of the most relevant studies did not, however, specifically report differences in sweetness intensity between their defined sweet taste liker phenotypes, limiting meaningful contrasts between our findings and prior work.

### 4.4. Recommended Criteria for the Identification of Distinct Sweet Taste Liker Phenotypes

Except for a pilot experiment [27], this is the first study suggesting specific criteria for the identification of the distinct sweet taste liker phenotypes that could be considered as both statistically robust and easy-to-apply. One core element of the proposed simpler approach is the administration of a single sucrose concentration that allows for both a less time-consuming and resource-demanding assessment process and for elimination of potential issues from the contrast effects which are “hard-wired” to longer protocols [72]. Within the taste literature, this in a not a novel concept. In 1980, Lawless addressed the need to identify an efficient classification method that could be used to rapidly screen large cohorts in terms of bitter taste phenotypes for phenylthiocarbamide (PTC) and 6-n-propylthiouracil (PROP), i.e., thiourea tasters and nontasters [73]. After using multiple approaches within the same study cohort, he concluded intensity ratings (on a 7-point scale) for a single antimodal concentration of PTC or PROP presented in a two-series taste test allowed for a rapid and reliable separation of the tasters from the nontasters [73].

Despite using a similar analysis to that of Asao et al. [27], we concluded that approximately twice the concentration of sucrose, compared to the concentration they proposed, is required to deliver the highest sensitivity and specificity in the discrimination between distinct sweet taste liker phenotypes. A small sample size, dichotomous grouping, and participants’ pre-test fasting state in the earlier pilot experiment [27] raise questions about the broader applicability of the concentration (0.598 M sucrose) recommended in their study. Indeed, other studies using multiple sweet taste stimuli identified concentrations ranging from 0.83 M (e.g., in References [66,74,75,76,77,78]) to 0.99 M (e.g., in References [79,80,81]). Moreover, the 0.6 M sucrose solution referred in Tuorila et al. [23] was actually shortlisted from their previous work where two additional lower concentrations were tested but not any higher [82]. Finally, the replication in our sample of the proposed by Asao and colleagues’ U-shaped association between sucrose concentration and reproducibility of the liking ratings across the repeated blocks of the taste test [27] may also bear critically upon sweet-liking protocols based on intermediate concentrations. Indeed, taste measures for about 40% of the adult sample in Garneau et al. [13] indicated indifferent responses to a range of stimuli between 0 M and 0.4 M sucrose.

Considering the comparatively less sophisticated and less restrictive concepts of the VAS compared to the labelled magnitude or Likert-type scales, the decision to record liking on an analogue scale further strengthens our classification criteria proposal. In particular, VAS-based ratings are independent of the range of prior sensory experiences and of the assumption that the same descriptors (labels) reflect equivalent meaning across different responders [83,84]. That said, in our lab, we have repeatedly observed that participants find VAS to be more straightforward than gLMS, although when we directly contrasted the two scales in a sample of young educated adults, VAS and gLMS yielded similar results [17]. Additionally, VAS is appropriate for recording the multi-dimensional continuum of human responses that a fixed pre-coded format does not by principle permit [85]. Clearly, no scaling approach is perfect: the “anchor effect” phenomenon (centering bias) characterized by less use of the extreme response has been associated with most rating scales, the VAS included [72]. Overall, we propose that utilizing VAS for sweet-liking assessment when phenotyping protocols are applied to groups of diverse characteristics is likely to come with the least challenges.

### 4.5. Controlling for Protocol Conditions

Although previous research presents an inconclusive picture [16,62,86], some studies report an effect of hunger [10,87,88] and thirst [89] on liking for sweet taste stimuli. It was thereby critical to ensure that recorded sensory responses were not driven by participants’ motivational state and that the motivational state did not differ between the contrasted sweet taste liker phenotypes. Analysis of the pre- and post-test levels of hunger and thirst across our study sample and between-groups confirmed this was not so.

The nature of changes in levels of hunger and thirst over the test period (increased and decreased by 15.2% and 10.1%, respectively) also indicated little or no likely influence of post-ingestive effects of sucrose on the sensory-related measures [90], suggesting the “sip and spit” protocol worked as expected. Notably, Running and Hayes [91] observed no significant differences in the rated intensity of a 0.5 M sucrose solution when “sip and spit” and “sip and swallow” protocols were contrasted. Nonetheless, the differences in the density of taste buds [92] and in the associated saliva [93] across the different regions of the oral cavity and the known role of gastrointestinal tract’s sweet taste receptors in metabolic regulation [94,95], suggest a need for both explicit instructions and subsequent compliance checks in sensory evaluations, particularly when a wide range of concentrations or a relatively strong solution are being tested.

### 4.6. No Effect of Sweet Taste Liker Phenotype on Participant Characteristics

Analysis of this young healthy sample found no effect of sweet taste liker phenotype on the few demographic, lifestyle, and anthropometric characteristics we examined. First, the frequency distribution of the SL phenotype did not differ between women and men. With the exception of the multi-ethnic cohort of Thai et al. [53], lack of sex differences in sweet-liking is consistent with previous published work focusing on sweet taste liker phenotypes generated from simple sucrose solution-based taste tests and where women and men were represented equally [27,49,52,64,66,77]. In his recent review, Spence [96] argues that individual differences rather than sex differences might be the most important influence on shaping our taste worlds, particularly when the hedonic aspects of taste are studied. Animal models provide equivocal results on sucrose sensory properties by sex [97]. These findings fail to support Katz’s theory of “gendered eating patterns” generated by either evolution or, according to others, by cultural norms [98], as well as baseline reports from the NutriNet-Santé study where, remarkably, men and not women liked sweet tastes more [99]. It is worth stressing though that sensory data in the French cohort were collected indirectly using “Pref-Quest,” a proxy of laboratory-based taste tests that measures recalled liking for different taste modalities via asking questions on selective food items and eating habits [100]. In the present work, we also failed to observe an effect of age on hedonic responses to sweet taste. This stands in direct contrast to the fairly consistent effect of age on sweet-liking whenever children or adolescents were compared with adult populations [101,102,103,104], and may be due to the relatively restricted age range tested here. To note, in some [13,16,74,76,78,105,106,107,108] but not all [13,81,109,110,111] studies testing middle-aged or older adults, SDs and those with an inverted U-shaped response outnumbered SLs. Critically, methodological limitations that may lead to possible overestimation of the SD phenotype in prior studies cannot also be overlooked [11].

Other factors worth exploring with regard to humans’ responses to sweet taste are dieting and BMI. Regarding attempts to investigate how being on a weight loss diet affects classification into the distinct sweet taste liker phenotypes, evidence has been loose and is drawn on research on sweet-liking either as a continuous measure (e.g., in References [112,113,114]) or assessed via questionnaires instead of laboratory-based taste tests [99]. As discussed in a recent review, bariatric surgery is also likely to augment gustatory sensitivity to sweet taste and to attenuate relevant hedonic responses post-operatively [115]. In our study, being a former dieter was more apparent in SDs. This may seem counterintuitive to the sensory specific satiety theory (decline in pleasantness for a food stimulus subsequent to consumption compared with the uneaten [29]), but could be backed up within the hedonic hunger context (motivation to consume palatable foods in the absence of food deprivation [116]). Nonetheless, no explicit information on the timing, duration, or mode of the dietary regime or the extent of weight loss and weight regain was collected. Additionally, considering the small size of this particular subgroup and the subsequent lack of significance, caution is advised in interpreting this observation until replicated. BMI, on the other hand, did not differ across the three sweet taste liker phenotypes. Although one can argue that this was due to the limited range of BMI in our sample, our finding was consistent with a sizable body of published evidence [13,14,15,17,24,49,53,66,69,76,106,117,118,119]. It is also of note that some early reports testing individuals of greater BMIs showed that obese were more often classified into the SD phenotype than normal-weight participants [16,26,54,120,121]; only one study of 12 participants has provided suggestive evidence for the opposite association [25].

### 4.7. Potential Mechanisms

Different mechanisms may account for the observed variations in affective responses to sweet taste, and fundamental biology likely plays a part. Sweet tasting substances activate various neural circuits including some associated with dopamine-linked reward centers in the prefrontal cortex [122,123,124]. This activation accommodates the urge to meet physiological needs such as the central nervous system’s energy supply (e.g., in Reference [125]). Internal state-specific factors (“homeostasis”) have also been implicated in explaining the variation of hedonic responses to sweet taste as a function of deprivation state. In this context enhanced sweet-liking in fetuses [126,127] and infants [128,129,130] may relate to the increased needs for energy during the stages of rapid growth [131]. Likewise, Coldwell and colleagues reported that SL adolescents had higher levels of a bone growth factor compared with their SD peers [49]. Similarly, negative gustatory alliesthesia, which refers to diminishing liking as a response to internal energy abundance (as in satiety or obesity) [36], has been proposed to contribute to the apparent inverse relationship between BMI and sweet-liking.

Later advances have implicated taste genetics with sweetness, both directly and indirectly. TAS1R2 and TAS1R3 taste receptor genes have directly been linked to sweet taste perception [132,133,134]. The heterodimeric protein encoded by these genes is expressed in taste receptor cells in the oral cavity, providing the mechanism by which sweet taste occurs [135]; subsequently, these receptors have also been found in extra oral tissues [123]. Salivary glucose levels and salivary protein profile have recently identified as additional potential determinants of sweet taste perception [136]. Finally, some reports suggest that differences in the density of structures that house taste cells (i.e., fungiform papillae) may explain differences in suprathreshold taste intensity, including sweetness [92,137], although others account conflict with this explanation [138,139,140,141].

### 4.8. Limitations

The present study has some limitations that require further confirmatory analyses in different populations to allow the proposed method to be applied universally. First, we had a gender-imbalanced sample of young adults primarily from European Caucasian ancestry. Past literature has partly identified more SLs than SDs when direct contrasts between younger and older adults were performed [16,26,47,77]. Whether sweet taste liker phenotypes vary by ethnic group is, however, not yet well understood [18,23,49,53,76,107]. Nevertheless, due to the higher risk of many non-Caucasian ethnic groups and of older versus much younger individuals in developed countries for non-communicable diseases [142], this research area is worthy of further investigation. Our findings may also not translate to populations with a different habitual intake of sugar. Studies in the U.S., for example, suggest a slightly higher daily intake of free sugars [143] compared with U.K.-based cohorts [144], whereas the recommended daily allowance [145] is also double the U.K. recommendations [146]. On the basis of the conflicting evidence surrounding the influence of exposure in sweet-tasting foods on hedonic responses to sweetness [147,148], this limitation may leave particular populations vulnerable to any possible interplay between sweet-liking patterns and eating patterns and therefore much still need to be learned. Moreover, women and men in our sample were not of a representative BMI for their age-matched group [46]. Whilst this is presumably a caveat for the generalizability of our results, the reader is advised to consider that, as noted earlier, both in our study and elsewhere, BMI did not differ by sweet taste liker phenotype. Still, the fact that the observed proportion of SDs was relatively low, although it was expected from phenotyping results from prior studies using HCA (see Section 4.3 for details), it also means that group contrasts need to be treated with some caution. Finally, no phenotyping method is beyond limitations. The one inherent in using HCA is the lack of a formal “stopping rule” in the clustering process; the researcher is called to indicate the number of stages displayed in the agglomeration schedule that need to be eliminated from further merging and then manually incorporate this decision on the generated dendogram [41].

## 5. Conclusions

The present study confirms that the expression of sweet-liking is not universal but responses to sweet taste stimuli vary considerably across people. What is new is the statistical determination of some robust but concurrently usable classification criteria for the identification of the different sweet taste liker phenotypes in a large-scale study. Despite limitations arising mainly from participant characteristics, there is good reason to believe that our approach might still be widely applicable as HCA-based liking patterns between our U.K. based study and those by American [13] and Korean [9] researchers largely align. Conceivably, the potential of a broader use of the psychophysical comparisons we delivered herein in epidemiological studies and clinical trials could have a fruitful impact on research associated with health and wellbeing. Accordingly, we may now have appropriate tools to finally address a longstanding issue first Mattes noted over 30 years ago, that is: “The question remains whether individual responsiveness to sweet taste can tell us anything about the individual, his or her physiological or nutritional status, or the likely patterns of food selection.” [149].

## Figures and Tables

**Table 1:** Participant characteristics.

	Total	Sweet Taste Like Phenotype ^1,2^		
		Sweet Liker	Inverted U-Shaped	Sweet Disliker
	*n* = 148	*n* = 46	*n* = 73	*n* = 27
Gender, N (%)				
Woman	105 (70.9)	33 (71.7)	48 (65.8)	22 (81.5)
Man	43 (29.1)	13 (28.3)	25 (34.2)	5 (18.5)
Ethnicity, N (%)				
Caucasian	112 (75.7)	39 (84.8)	53 (72.6)	19 (70.4)
Asian	14 (9.4)	2 (4.3)	9 (12.3)	3 (11.1)
Other	22 (14.9)	5 (10.9)	11 (15.1)	5 (18.5)
Dieting, N (%)				
Once or more times in the past	52 (35.6)	15 (32.6)	23 (31.9)	12 (46.2)
Never	94 (64.4)	31 (67.4)	49 (68.1)	14 (53.8)
Added sugar in drinks/cereals, N (%)				
More when being younger	72 (48.6)	18 (39.1)	39 (53.4)	14 (51.9)
Same as when being younger	27 (18.2)	11 (23.9)	9 (12.3)	7 (25.9)
Never	49 (33.1)	17 (37.0)	25 (34.3)	6 (22.2)
Age range (median) in years	18.2–34.0 (20.2)	18.3–32.8 (19.8)	18.2–34.0 (20.2)	18.2–34.0 (20.9)
BMI range (median) in kg/m^2^	17.8–32.4 (22.1)	17.9–29.1 (23.0)	17.8–32.4 (21.6)	18.2–30.3 (22.7)

BMI, body mass index; Q1, 25th percentile; Q3, 75th percentile. All frequencies reported refer to valid percentages. ^1^ Participants demonstrating erratic responses to sweet stimuli (*n* = 2) were excluded from this analysis. ^2^ *p* > 0.05 for all between group comparisons performed with chi-square or Kruskal Wallis tests.

**Table 2:** Sensitivity and specificity checks to discriminate sweet dislikers (cluster 3) from the rest of sweet taste liker phenotypes.

Liking Cut-Off Scores	Sucrose Concentration (M)							
	0.25		0.5		0.67		1.0	
	Sensitivity (%)	Specificity (%)	Sensitivity (%)	Specificity (%)	Sensitivity (%)	Specificity (%)	Sensitivity (%)	Specificity (%)
−20	13.6	100.0	36.4	100.0	45.5	99.1	81.8	96.5
−15	13.6	100.0	54.5	97.4	68.2	95.6	90.9 *	93.9 *
−10	27.3	99.1	63.6	94.7	77.3	92.1	95.5	87.7
−5	50.0	94.7	77.3	93.0	95.5	86.0	100.0	77.2
0	59.1	89.5	90.9	86.8	100.0	76.3	100.0	68.4

Percentages (%) with an asterisk (*) indicate the dyad of sucrose concentration and liking cut-off score with the highest combined sensitivity and specificity for the prediction of the sweet disliker phenotype across all dyads tested.

**Table 3:** Sensitivity and specificity checks to discriminate sweet likers (cluster 1) from the rest of sweet taste liker phenotypes.

Liking Cut-off Scores	Sucrose Concentration (M)							
	0.25		0.5		0.67		1.0	
	Sensitivity (%)	Specificity (%)	Sensitivity (%)	Specificity (%)	Sensitivity (%)	Specificity (%)	Sensitivity (%)	Specificity (%)
0	95.5	26.1	100.0	40.2	100.0	55.4	100.0	64.1
5	79.5	43.5	100.0	54.3	97.7	63.0	100.0	77.2
10	56.8	67.4	100.0	67.4	97.7	76.1	97.7	89.1
15	38.6	84.8	88.6	79.3	88.6	87.0	97.7 *	93.5 *
20	20.5	88.0	63.6	87.0	79.5	96.7	84.1	97.8

Percentages (%) with an asterisk (*) indicate the dyad of sucrose concentration and liking cut-off score with the highest combined sensitivity and specificity for the prediction of the sweet liker phenotype across all dyads tested.
